# Identification of ZBTB4 as an immunological biomarker that can inhibit the proliferation and invasion of pancreatic cancer

**DOI:** 10.1186/s12885-023-10749-x

**Published:** 2023-03-22

**Authors:** Zhe Yang, Feiran Chen, Feng Wang, Xiubing Chen, Biaolin Zheng, Xiaomin Liao, Zhejun Deng, Xianxian Ruan, Jing Ning, Qing Li, Haixing Jiang, Shanyu Qin

**Affiliations:** Department of Gastroenterology, The First Affiliated Hospital of Guangxi Medical University, No 6 Shuangyong Road Nanning, Guangxi Zhuang, Autonomous Region People’s Republic of China

**Keywords:** ZBTB4, Immunological biomarker, Pancreatic cancer, Tumor microenvironment

## Abstract

**Background:**

Zinc finger and BTB domain-containing protein 4 (ZBTB4) belongs to the zinc finger protein family, which has a role in regulating epigenetic inheritance and is associated with cell differentiation and proliferation. Previous studies have identified aberrant ZBTB4 expression in cancer and its ability to modulate disease progression, but studies on the immune microenvironment, immunotherapy and its role in cancer are still lacking.

**Methods:**

Human pan-cancer and normal tissue transcriptome data were obtained from The Cancer Genome Atlas. The pan-cancer genomic alteration landscape of ZBTB4 was investigated with the online tool. The Kaplan–Meier method was used to evaluate the prognostic significance of ZBTB4 in pancreatic cancer. In parallel, ZBTB4 interacting molecules and potential functions were analyzed by co-expression and the correlation between ZBTB4 and immune cell infiltration, immune modulatory cells and efficacy of immune checkpoint therapy was explored. Next, we retrieved the Gene Expression Omnibus database expression datasets of ZBTB4 and investigated ZBTB4 expression and clinical significance in pancreatic cancer by immunohistochemical staining experiments. Finally, cell experiments were performed to investigate changes in pancreatic cancer cell proliferation, migration and invasion following overexpression and knockdown of ZBTB4.

**Findings:**

ZBTB4 showed loss of expression in the majority of tumors and possessed the ability to predict cancer prognosis. ZBTB4 was closely related to the tumor immune microenvironment, immune cell infiltration and immunotherapy efficacy. ZBTB4 had good diagnostic performance for pancreatic cancer in the clinic, and ZBTB4 protein expression was lost in pancreatic cancer tumor tissues. Cell experiments revealed that overexpression of ZBTB4 inhibited the proliferation, migration and invasion of pancreatic cancer cells, while silencing ZBTB4 showed the opposite effect.

**Conclusions:**

According to our results, ZBTB4 is present in pancreatic cancer with aberrant expression and is associated with an altered immune microenvironment. We show that ZBTB4 is a promising marker for cancer immunotherapy and cancer prognosis and has the potential to influence pancreatic cancer progression.

**Supplementary Information:**

The online version contains supplementary material available at 10.1186/s12885-023-10749-x.

## Introduction

Cancer places a considerable clinical and financial burden on public health. Morbidity and mortality also remain a problem for advanced cancer patients. Despite advances in management and diagnosis, rates of survival have not improved significantly [1]. Therefore, in-depth insights into the molecular mechanisms that contribute to cancer progression are needed for the development of novel therapeutic targets and the improvement of current strategies to cure the disease. The Cancer Genome Atlas (TCGA) and the Gene Expression Omnibus (GEO) database provide a common platform for the study of multiple cancer types, with a sizable number of cases being sequenced at the whole-genome level [2, 3]. With this platform, target genes can be explored to understand the similarities and differences in their expression levels in various human tumors.

ZBTB4, also known as ZNF903 or KAISO-L1, belongs to the C_2_H_2_ zinc finger protein family, which contains the C_2_H_2_ zinc finger and a BTB/POZ domain. ZBTB4 has roles in cell differentiation and proliferation. Researchers have found that ZBTB4 can affect the immune microenvironment and it has the ability to regulate the secretion of certain immune factors [4]. ZBTB4 is a mammalian epigenetic regulator with high affinity for methylated CpG. Decreased expression of ZBTB4 is related to high instability of the genome; thus, ZBTB4 plays an important role in maintaining the stability of mammalian genomes [5, 6]. The expression of ZBTB4 is downregulated in colon cancer, gastric cancer, retinoblastoma, Ewing sarcoma, breast cancer and prostate cancer, suggesting that ZBTB4 may have the potential to inhibit tumor development [7–13]. ZBTB4 also affects Epithelial-mesenchymal transition (EMT) in lung cancer [14]. At present, the role of ZBTB4 in various types of tumors is still unclear. In particular, the immunological and biological role of ZBTB4 in tumors requires elucidation as research is lacking.

The characteristic histopathology of tumors includes poorly differentiated cancer cells embedded in a connective tissue hyperplasia matrix, which is composed of immune cells, cancer-related fibroblasts and extracellular matrix. However, how various cell types in the tumor microenvironment interact to influence tumor progression, metastasis and therapeutic response has not been clarified. At present, some studies have found that various cell components in the tumor microenvironment can affect the survival of specific cells [15, 16]. The composition of tumor infiltrating immune cells can be predicted by calculations from large sets of tumor RNA sequencing data, and immune cell infiltration is also closely related to the efficacy of immunotherapy [17]. Therefore, the efficacy of immunotherapy can be predicted according to the gene expression data of specific immune molecules.

In this study, we used TCGA and GEO RNA sequencing data to study the expression trend of ZBTB4 in various types of tumors. In the tumors with abnormal expression of ZBTB4, the influence of ZBTB4 on the prognosis of patients was analyzed using follow-up data of survival period. In addition, we explored the relationship between ZBTB4 expression and tumor immune cell infiltration, tumor mutational burden (TMB), microsatellite instability (MSI) and multiple molecular signal pathways. Importantly, we verified the expression of ZBTB4 in clinical specimens of pancreatic cancer by immunohistochemistry and verified the expression of ZBTB4 in pancreatic cancer cell lines in experiments to explore its biological functions. Our findings reveal the difference in the expression of ZBTB4 in various tumors and its interaction with the immune microenvironment. We also show that the expression of ZBTB4 is downregulated in pancreatic cancer cell lines and can inhibit their development. As a tumor suppressor, ZBTB4 can affect the tumor immune microenvironment, and may be a potential tumor immunotherapy target.

## Materials and methods

### Pan-cancer datasets and processing

We obtained the pan-cancer datasets (TCGA Pan-Cancer; *N* = 10,535, G = 60,499) from the University of California Santa Cruz (UCSC) database (https://xenabrowser.net/) [18]. The dataset has been subjected to Transcripts Per Kilobase per Million mapped reads (TPM) normalization. We subsequently extracted the expression data of the ZBTB4 gene from each dataset, and each expression value was log_2_ (TPM + 1) transformed. We excluded cancers with less than three samples, and finally obtained expression data for 26 cancers. Cancer abbreviations are shown in Supplementary materials (Supplementary Table S1). We used the online tool provided by the SangerBox 3.0 website (http://vip.sangerbox.com/) for data visualization [19].

### Analysis of genomic mutations in human cancers

We retrieved human cancer genomic mutation data from the cBioPortal database, which contains information on proteomics, gene expression and genetics in human cancers [20]. We used the Real-time tool on the cBioPortal website (http://www.cbioportal.org) to map the ZBTB4 gene mutation bars in human cancers and show the frequency of gene alterations including the following: mutations, structural variants, amplifications, deep deletions and multiple alterations.

### Prognostic analysis

Prognostic information included overall survival and disease-specific survival. All prognostic information was downloaded from the UCSC database using the Kaplan–Meier model and univariate Cox regression analysis was used to calculate the prognostic role of ZBTB4 in various cancers. The R package maxstat (maximally selected rank statistics with several p-value approximations version 0.7–25) was used to calculate the optimal cut-off value for sample grouping by setting the minimum grouping sample size greater than 25% and the maximum sample size grouping less than 75% to obtain the optimal cut-off value. Patients were divided into high and low groups based on this value. The prognostic differences between the two groups were further analyzed using the survfit function of the R package survival. P-values and 95% confidence intervals and their hazard ratios were obtained using the Log-rank test method to compute the prognostic differences between the two sample groups.

### Immune cell infiltration analysis

Tumor Immune Estimation Resource (TIMER, https://cistrome.shinyapps.io/timer/) is a website that enables users to analyze the level of immune cell infiltration in cancer tissues from gene transcriptome data [21]. We used the TIMER2.0 database to compute the correlation of ZBTB4 expression with 21 types of immunomodulatory cells, including cancer-associated fibroblasts, progenitors of lymphoid, B cells, neutrophils, hematopoietic stem cells, CD4^+^ T cells, progenitors of myeloid, progenitors of monocytes, endothelial cells, eosinophils, regulatory T cells, T cell follicular helper, natural killer T cells, γ/δ T cells, monocytes, macrophages, dendritic cells, CD8^+^ T cells, mast cells and natural killer cells.

### Analysis of immunomodulation-related genes, tumor mutational burden and microsatellite instability

The expression of five types of immunomodulatory-related genes (chemokine, receptor, major histocompatibility complex, immunoinhibitor and immunostimulator) and ZBTB4 were extracted from the pan-cancer dataset. Pearson correlations between of these genes were calculated using the online tool provided by SangerBox. In parallel, the tool was used to analyze the correlation of ZBTB4 expression with TMB and MSI.

### Molecular interaction network construction and functional enrichment analyses

The GeneMANIA database (http://genemania.org/) enabled us to retrieve functionally similar genes by searching for the interacting molecules [22]. Gene Ontology and Kyoto Encyclopedia of Genes and Genomes[23–25] enrichment analyses were used to analyze genes that were functionally similar to ZBTB4, and data visualization was performed using the SangerBox.

### Expression of ZBTB4 and correlation analysis of clinical characteristics

The GSE125158 dataset was downloaded from the GEO database and receiver operating characteristic (ROC) curve analysis was performed. The pancreatic cancer tissue microarray (ZL-PanA961) used for immunohistochemical staining and its clinical characteristics information were provided by Well Biotech. The ZBTB4 antibody (D263134, Sangon, Shanghai, China) was used with 1:50 dilution. Histochemistry score was assessed on immunohistochemically stained images using Visiopharm software, and statistical significance between the histochemistry score of pancreatic ductal adenocarcinoma and adjacent tissues was calculated using paired t-tests. Correlation of clinical characteristics information was calculated by chi-square test using SPSS25.0 software.

### Cell culture and intervention of ZBTB4 expression

Cell lines HPDE6-C7, Panc-1, Bxpc-3, Aspc-1 and Cfpac-1 were purchased from Shanghai Institute of Biochemistry and Cellular Science, Chinese Academy of Sciences. The cells were cultured in a constant temperature cell culture incubator at 37℃ with 5% CO_2_. The medium contained 10% fetal bovine serum and 1% penicillin and streptomycin. Plasmids for ZBTB4 overexpression were supplied by MiaoLingPlasmid, and the plots are shown in the Supplementary Materials (Supplementary Figure S1). Small interfering RNA for silencing ZBTB4 was purchased from GenePharma, and the sequence is shown in the Supplementary Materials (Supplementary Table S2). Cells were transfected using Lipo8000™ Transfection Reagent (C0533, Beyotime, Shanghai, China) at a cell confluence of approximately 70–80%.

### Western blot analysis

The cells were lysed in 1 × RIPA buffer containing 1 mM phenyl methyl sulfonyl fluoride (PMSF), and the protein concentration was quantified by the Bradford protein assay. A total of 30 μg protein lysates from each sample were separated in 10% SDS-PAGE and then transferred onto polyvinylidene fluoride membranes (Millipore, Billerica, MA, USA). After blocking with 5% non-fat milk, the membranes were incubated overnight on ice with the primary antibodies against ZBTB4 (sc-514883, Santa Cruz Biotechnology, CA, USA) and GAPDH (all with 1:1000 dilution). After washing, the membranes were probed with a goat anti-mouse IgG (H + L) secondary antibody with a fluorescent dye solution in 4 × PEG (DyLight 800, SA5-35,521, ThermoFisher, MA, USA). The images were analyzed with ImageJ software for grayscale values.

### Quantitative real-time polymerase chain reaction

The total RNA of the cells (HPDE6-C7, Panc-1, Bxpc-3, Aspc-1, and Cfpac-1) was extracted by TRIZOL reagent (TaKaRa, Japan). The RNA samples were reverse transcribed into cDNA using a PerfectStart Uni qPCR + RT kit (AUQ-01, TransGen Biotech Co, Beijing, China). The primer sequences used were as follows: ZBTB4 forward 5′-ACTGTGTCAGATCACTGTGCGAATAG-3′; ZBTB4 reverse 5′-CGTCCTCCTCACTCTCCTCCATC-3′; GAPDH forward 5′-GCACCGTCAAGGCTGAGAAC-3′; and GAPDH reverse 5′-TGGTGAAGACGCCAGTGGA-3′. ZBTB4 expression was determined by quantitative real-time PCR using Power SYBR Green PCR master mix (AUQ-01, TransGen Biotech Co, Beijing, China). Relative mRNA expression levels were calculated by the Ct method, and GAPDH was chosen as the control. Each sample was run in triplicate.

### CCK8 detection

Cells were digested 24 h after transfection, then 5 × 10^3^ cells were counted and inoculated into 96-well plates with at least five replicate wells per group and placed in a constant temperature cell culture incubator with 5% CO_2_ at 37 °C. Cells were assayed at 0 h, 24 h, 48 h, and 72 h using the CCK8 kit (BS350, Biosharp, Anhui, China).

### EdU assays

EdU assays were performed using the BeyoClick™ EdU Cell Proliferation Kit with Alexa Fluor 488 (C0071S, Beyotime, Shanghai, China). Cells were fixed using 4% paraformaldehyde 48 h after transfection and subsequently assayed according to the kit’s user guide.

### Transwell migration and invasion assay

A 24-well, 8-μm Transwell chamber (3422, Corning Costar, NY, USA) coated with or without matrix gel was prepared in advance. Next, 24 h after transfection of cells, 200 µl of cell suspension containing 8 × 10^4^ cells was inoculated into the upper chamber and 600 µl of medium containing 20% fetal bovine serum was added to the lower chamber. The chambers were washed with PBS and fixed with 4% paraformaldehyde for 30 min, followed by staining with 0.1% crystal violet for 5 min. Finally, the cells were photographed for cell counting.

### Statistical analysis

Unpaired Student's t-Test analysis was used to calculate expression differences between normal and tumor samples in the pan-cancer data. Kaplan–Meier method (log-rank test) was used to assess the prognostic role of ZBTB4 expression in each cancer. Spearman correlation analysis was used to assess the statistical relationship between ZBTB4 and other factors, such as immune cell infiltration level, immunomodulatory genes, TMB and MSI. All statistical analyses were run in R statistical software (version 3.6.4) and SPSS statistical software (version 25.0). P values less than 0.05 were considered to be statistically significant.

## Results

### The abnormal expression of ZBTB4 in different cancer types

**Fig. 1:**
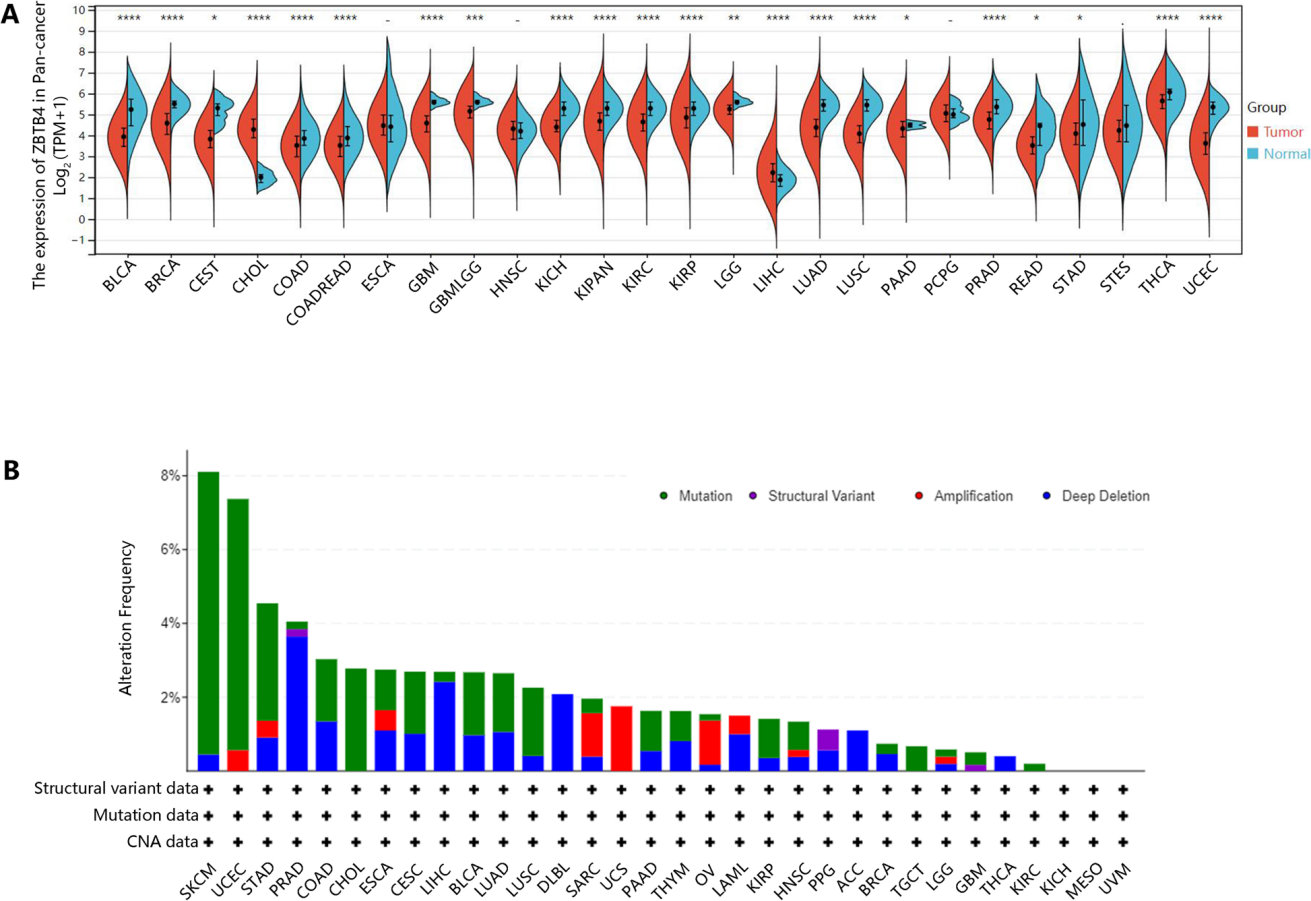
ZBTB4 expression in pan-cancer samples. **A** Expression of ZBTB4 in human pan-cancer samples based on The Cancer Genome Atlas database. **B** Mutation analysis of the ZBTB4 gene in human pan-cancer samples, based on the cBioPortal database, included structural variant data, mutation data and copy number alteration (CNA) data. ns = *p* > 0.05, *p < 0.05, **p < 0.01, ***p < 0.001, ****p < 0.0001

Data from TCGA pan-cancer were analyzed for ZBTB4 expression. We found reduced expression of ZBTB4 at mRNA levels in the following datasets: BLCA, BRCA, CESC, COAD, GBM, KICH, KIRC, LGG, LUAD, LUSC, PAAD, PRAD, READ, STAD, THCA and UCEC. Concomitantly, the expression of ZBTB4 was also markedly decreased in CHOL and LIHC. Based on our preliminary observations, the expression of ZBTB4 was decreased in most types of tumors. From these observations, we hypothesized whether a genetic mutation of ZBTB4 caused this phenomenon. The results are shown in Fig. 1A. We examined whether the ZBTB4 gene was altered in the TCGA pan-cancer samples. As shown in Fig. 1B, a higher mutation frequency (7.66%) was observed in SKCM, while the most “deep deletion” changes were observed in PRAD.

### The prognostic value of ZBTB4 in different cancer types

**Fig. 2:**
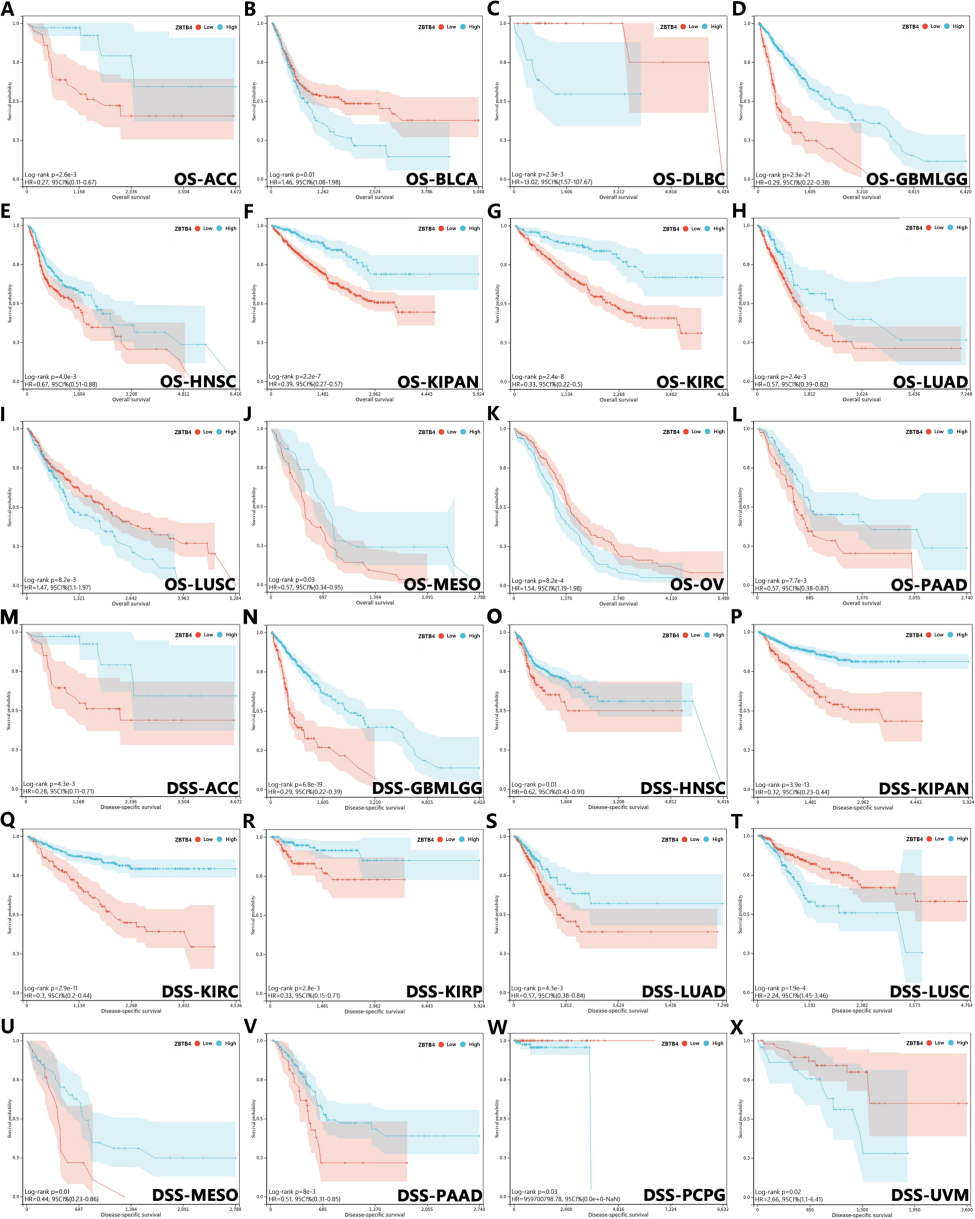
Survival analysis of ZBTB4 expression based on The Cancer Genome Atlas database. A–L ZBTB4 expression in ACC (**A**), BLCA (**B**), DLBC (**C**), GBMLGG (**D**), HNSC (**E**), KIPAN (**F**), KIRC (**G**), LUAD (**H**), LUSC (**I**), MESO (**J**), OV (**K**) and PAAD (**L**) was analyzed using the Kaplan–Meier method in relation to overall survival (OS). (M–X) The relationship between ZBTB4 expression and disease-specific survival (DSS) in ACC (**M**), GBMLGG (**N**), HNSC (**O**), KIPAN (**P**), KIRC (**Q**), KIRP (**R**), LUAD (**S**), LUSC (**T**), MESO (**U**), PAAD (**V**), PCPG (**W**) and UVM (**X**) was subsequently analyzed using the Kaplan–Meier method as well. Red is low expression and blue is high expression

For the TCGA Pan-Cancer data, we analyzed the correlation between the abnormal expression of ZBTB4 and the survival of patients by Kaplan–Meier curves. Representative data are shown in Fig. 2. In ACC, PAAD, MESO, KIRC, HNSC, KIPAN, LUAD and GBMLGG, low expression of ZBTB4 was significantly associated with shorter overall survival (Fig. 2A, D, E, F, G, H, J and L). In sharp contrast, high ZBTB4 expression in BLCA, DLBC, LUSC and OV was associated with poor overall survival (Fig. 2B, C, I and K). To further explore these features, we observed the relationship between ZBTB4 expression and disease-specific survival. The results showed that the loss of ZBTB4 in ACC, GBMLGG, HNSC, KIPAN, KIRC, KIRP, LUAD, MESO and PAAD could predict poor disease-specific survival (Fig. 2M, N, O, P, Q, R, S, U and V).

### The influence of ZBTB4 expression on tumor microenvironment

**Fig. 3:**
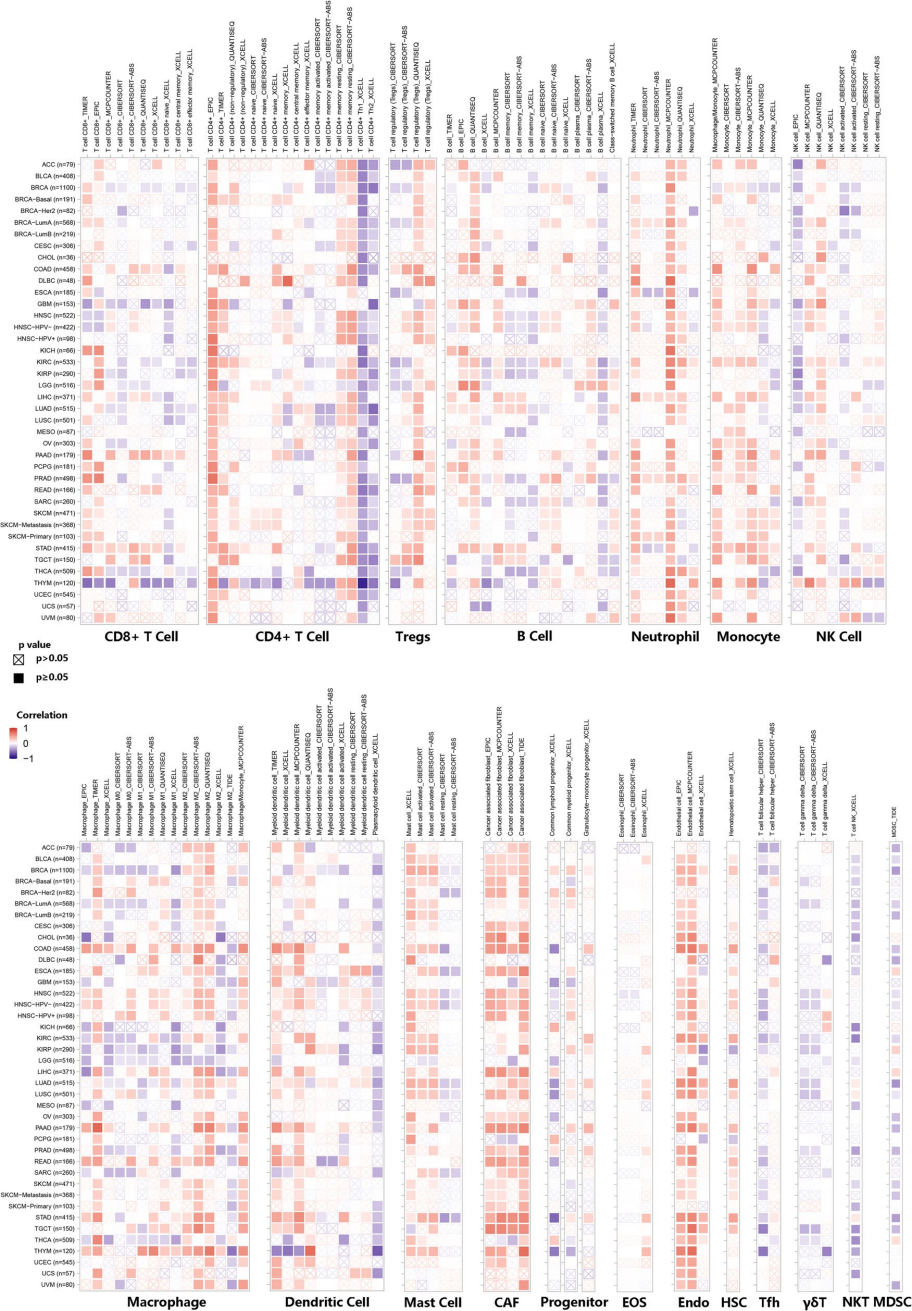
Correlation of ZBTB4 expression levels with 19 types of immunomodulatory-related cells, including CD8^+^ T cells, CD4^+^ T cells, regulatory T cells (Tregs),B Cell, Neutrophil, Monocyte, Natural killer(NK) Cell, Macrophage, Dendritic Cell, Mast Cell, Cancer-associated fibroblast (CAF), progenitors of lymphoid, progenitors of myeloid, eosinophil (Eos), endothelial cell (Endo), hematopoietic stem cell (HSC), T cell follicular helper (Tfh), T cell gamma delta (γ/δT), nature killer T cell (NKT), MDSC. Positive correlations are in red and negative correlations are in blue

Existing studies have demonstrated that the tumor microenvironment can influence tumor development and metastasis, and that this environment can enable tumors to evade immune surveillance. In addition, tumor microenvironment can affect patient prognosis and treatment choices. Here, we evaluated the relationship between ZBTB4 expression and immune cell infiltration with the TIMER database. The results indicated that the following cell types were correlated with ZBTB4: CD8^+^ T cells, CD4^+^ T cells, T cell regulatory, B cells, neutrophils, macrophages, monocytes, myeloid dendritic cells, nature killer T cells, mast cells, cancer-associated fibroblasts, common lymphoid progenitor, common myeloid progenitor, endothelial cell, eosinophil, granulocyte-monocyte progenitor, hematopoietic stem cells, T cell follicular helper, T cell natural killer and Myeloid-derived suppressor cells(MDSC) (Fig. 3). In conclusion, our findings demonstrate that ZBTB4 can modulate the tumor microenvironment.

### The correlation between ZBTB4 expression and immunomodulators, TMB and MSI

**Fig. 4:**
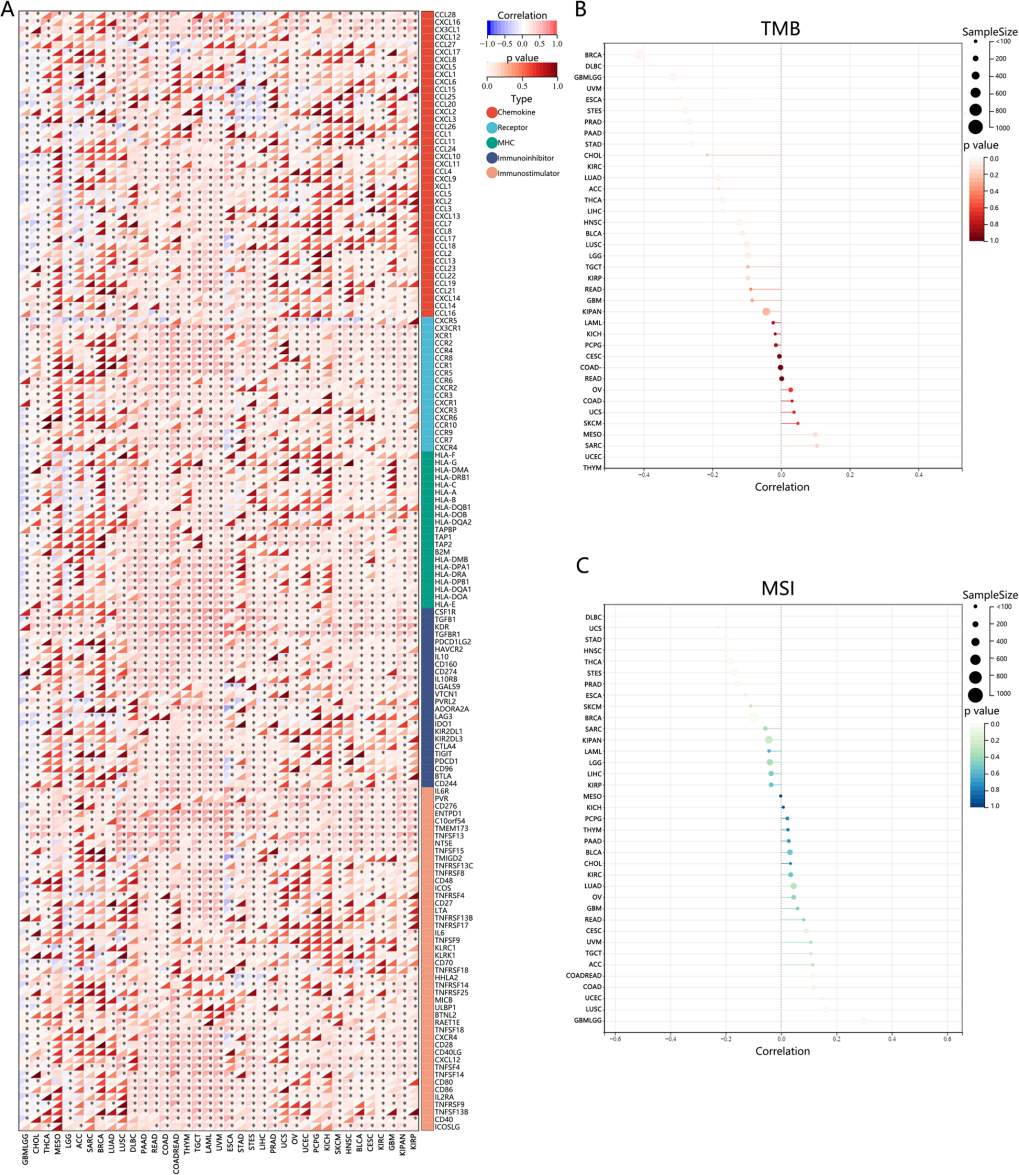
**A** Correlation analysis of ZBTB4 expression with five types of immunoregulatory genes. **B** Correlation analysis of ZBTB4 expression with tumor mutation burden in human pan-cancer. **C** Correlation analysis of ZBTB4 expression with microsatellite instability (MSI) in human pan-cancer. MHC, major histocompatibility complex

After acquiring the expression of ZBTB4, we performed correlation analysis by Spearman to identify the correlation of ZBTB4 expression with five classes of immunoregulatory-related genes. These genes included major histocompatibility complex, immunological stimulation, immunosuppression, chemokine, and chemokine receptor proteins. The results indicated that ZBTB4 showed a significant positive correlation with most of the immune-related genes in the remaining cancers except for GBMLGG (Fig. 4A). The current study demonstrated that TMB was strongly correlated with the efficacy response to immunotherapy, and we computed their Spearman correlation in each tumor and observed that ZBTB4 expression was significantly negatively correlated with TMB in 17 tumors (Fig 4B), including GBMLGG, LGG, LUAD, BRCA, ESCA, STES, STAD, PRAD, HNSC, KIRC, LUSC, LIHC, THCA, PAAD, UVM, BLCA and DLBC. MSI is associated with tumor development and often accompanied by DNA mismatch repair defects. In terms of the Spearman correlation, we observed that ZBTB4 expression was significantly positively correlated with MSI in five tumors, including GBMLGG, COAD, COADREAD, UCEC and LUSC (Fig. 4C). ZBTB4 expression was also significantly negatively correlated with MSI in seven tumors, including BRCA, STES, STAD, PRAD, HNSC, THCA and DLBC (Fig. 4C).

### The functional enrichment analysis and molecular interaction network of ZBTB4

**Fig. 5:**
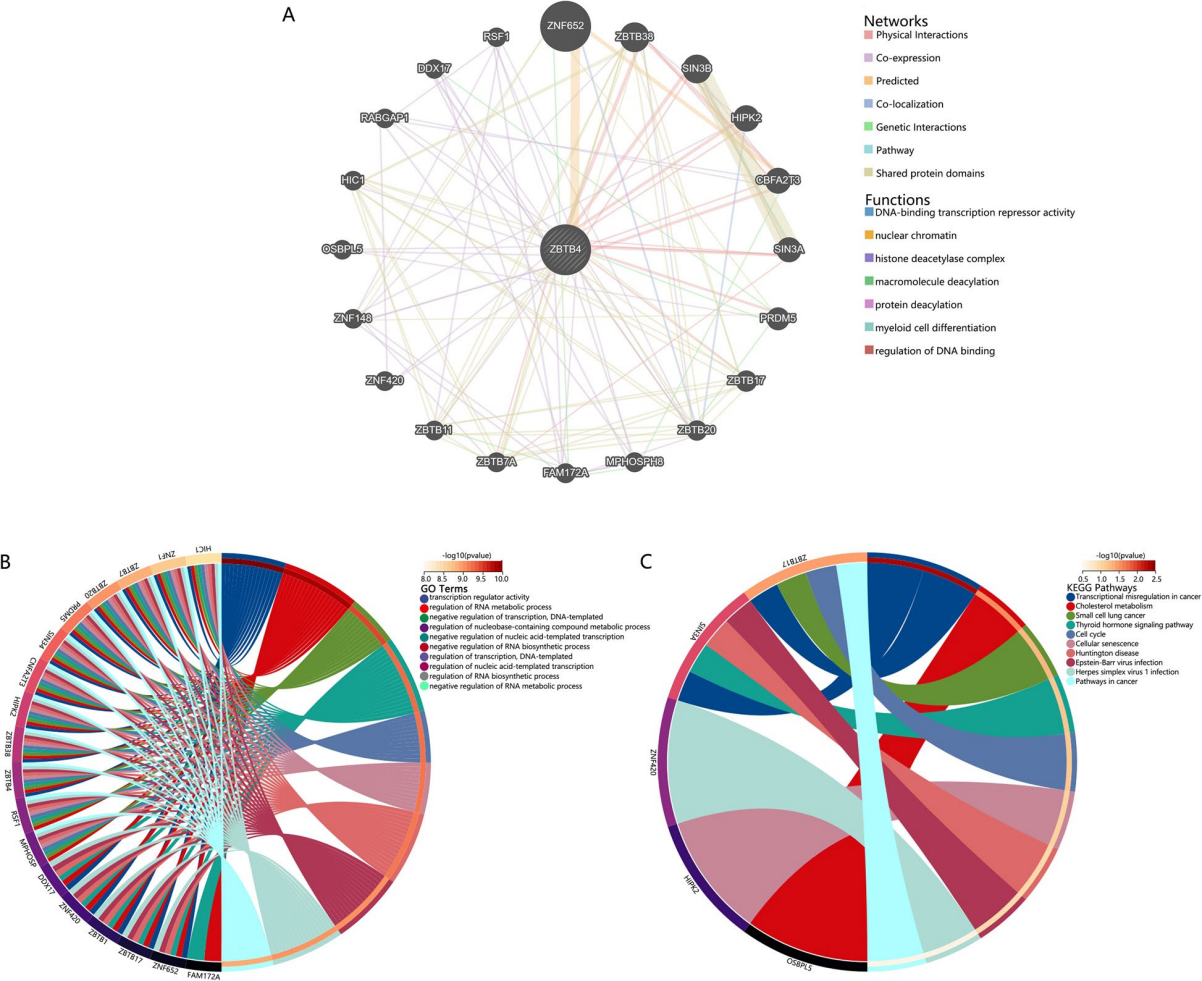
Potential functions and molecular interaction analysis of ZBTB4. **A** Network diagram of ZBTB4-interacting molecules using Gene MANIA. **B** The molecules interacting with ZBTB4 were subjected to Gene Ontology functional enrichment analysis. **C** Kyoto Encyclopedia of Genes and Genomes functional enrichment analysis of molecules interacting with ZBTB4

To further analyze the role played by ZBTB4 and its molecular mechanisms, we used Gene MANIA to analyze the molecules that may interact with it and selected 20 genes that may interact to map the interaction network; their main functions were focused on DNA-binding transcription repressor activity, nuclear chromatin, the histone deacetylase complex, macromolecule diacylation, protein diacylation, myeloid cell differentiation and regulation of DNA binding (Fig. 5A). Furthermore, we conducted enrichment analysis of these genes with Gene Ontology and the Kyoto Encyclopedia of Genes and Genomes resources. The results of Gene Ontology analysis indicated that the role of these gene sets are mainly the following: transcription regulator activity, regulation of RNA metabolic process, negative regulation of transcription, DNA-templated, regulation of nucleobase-containing compound metabolic process, negative regulation of nucleic acid-templated transcription, negative regulation of RNA biosynthetic process, regulation of transcription, regulation of nucleic acid-templated transcription, regulation of RNA biosynthetic process and negative regulation of RNA metabolic process (Fig. 5B). Results from the Kyoto Encyclopedia of Genes and Genomes dataset revealed that these genes were associated with the following: transcriptional mis regulation in cancer, cholesterol metabolism, small cell lung cancer, thyroid hormone signaling pathway, cell cycle, cellular senescence, Huntington disease, Epstein-Barr virus infection, Herpes simplex virus 1 infection and pathways in cancer (Fig. 5C).

### The clinical role of ZBTB4 in pancreatic cancer

**Fig. 6:**
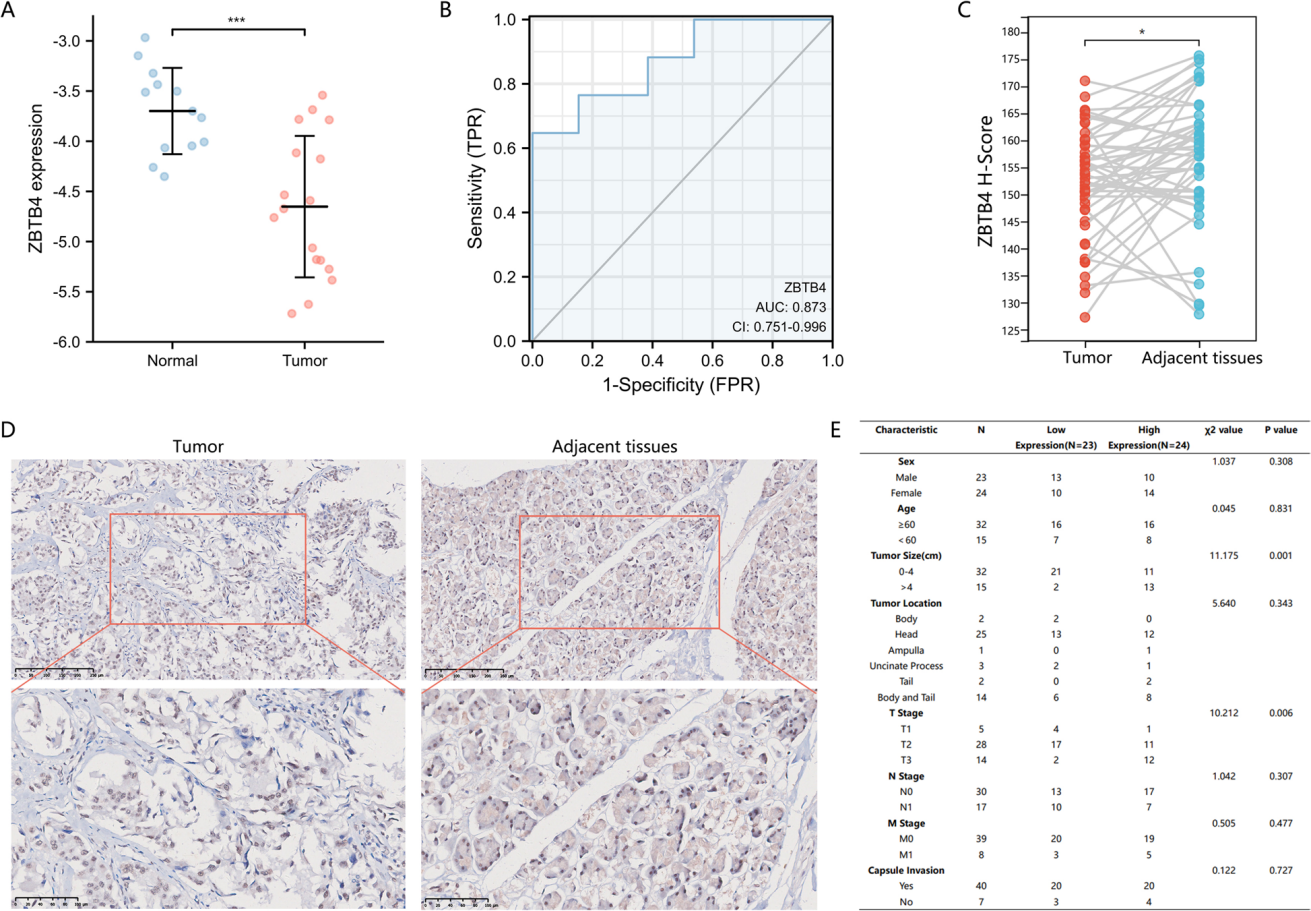
Clinical significance of ZBTB4 expression in pancreatic ductal adenocarcinoma. **A** ZBTB4 mRNA levels in peripheral blood of healthy individuals and pancreatic cancer patients in GSE125158. **B** Diagnostic ROC curve model drawn from GSE125158. Area under curve(AUC);True positive rate(TPR); False positive rate(FPR); Confidence interval(CI). **C** Pairwise analysis of ZBTB4 protein immunohistochemical staining scores. **D** Expression of ZBTB4 in pancreatic cancer tissue sections. **E** Correlation analysis of ZBTB4 expression with clinical characteristics of patients. ns = *p* > 0.05, *p < 0.05, ** *p* < 0.01, ***p < 0.001

This study reveals the important role of ZBTB4 in human pan-cancer. We are also concerned that the pan-cancer data identified a deletion of ZBTB4 expression in pancreatic cancer, which was also significantly associated with immune infiltration in pancreatic cancer tissues. Importantly, the lack of ZBTB4 expression in pancreatic cancer resulted in a poor prognosis. This makes it interesting to understand the role that ZBTB4 plays in pancreatic cancer. Here, we focused on the clinical role played by ZBTB4 in pancreatic cancer by searching the GSE125158 dataset in the GEO database. This was investigated using mRNA results from peripheral blood, which showed that ZBTB4 mRNA expression was remarkably reduced in the peripheral blood of pancreatic cancer patients relative to that in healthy individuals (Fig. 6A). The performance of ZBTB4 mRNA content in peripheral blood in the diagnosis of pancreatic cancer was evaluated using ROC diagnostic curves based on the expression levels; the ROC results indicated that ZBTB4 mRNA content in peripheral blood was of high diagnostic significance with an area under the ROC curve of 0.873 (Fig. 6B). We subsequently analyzed ZBTB4 protein expression in pancreatic cancer tissue microarrays using immunohistochemical staining and scored the staining intensity using the histochemistry score (Fig. 6C) and found that ZBTB4 protein was low in pancreatic cancer tissues after comparison with paired paraneoplastic tissues (Fig. 6D). Analysis of the patients’ clinical characteristics and ZBTB4 expression, as shown in Fig. 6E, demonstrated a correlation between ZBTB4 expression, tumor size and T Stage.

### The biological function of ZBTB4 in pancreatic cancer cell lines

**Fig. 7:**
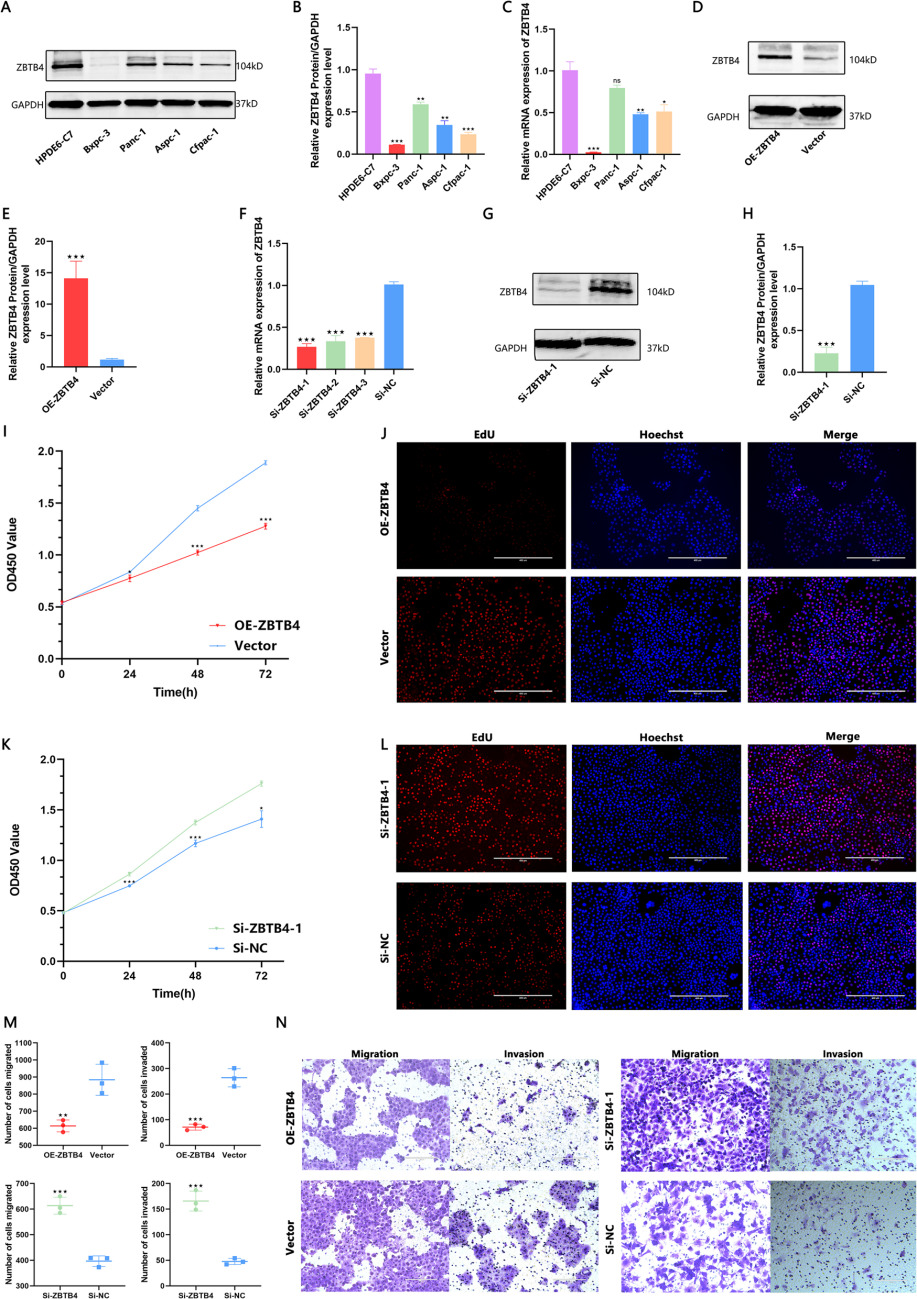
Function of ZBTB4 in pancreatic cancer cells. **A**, **B** Protein expression levels of ZBTB4 were detected in pancreatic cancer cell lines. **C** Detection of ZBTB4 mRNA content in pancreatic cancer cell lines. **D**, **E** Detection of the efficiency of overexpression of ZBTB4 in Bxpc-3 cells. **F**–**H** Detection of the efficiency of silencing ZBTB4 in Panc-1 cells. CCK8 (**I**) and EdU (**J**) detect the effect of overexpression of ZBTB4 on the proliferation of Bxpc-3 cells. CCK8 (**K**) and EdU (**L**) detect the effect of silencing ZBTB4 on the proliferation of Panc-1 cells. **M**, **N** Transwell assays were used to detect the effects of overexpression or silencing of ZBTB4 on the migration and invasion ability of Bxpc-3 and Panc-1 cells. The data shown in this figure are representative of three biological replicate experiments. ns = *p* > 0.05, *p < 0.05, ** *p* < 0.01, ***p < 0.001

The role of ZBTB4 in pancreatic cancer cells is not yet understood. First, we examined ZBTB4 protein and mRNA expression in human normal pancreatic ductal epithelial cells (HPDE6-C7) and four pancreatic cancer cell lines (Bxpc-3, Panc-1, Aspc-1 and Cfpac-1). Both RT-PCR and western blot results confirmed the low level of ZBTB4 in pancreatic cancer cell lines (Fig. 7A, B and C). Based on the expression level of each cell line, we selected Bxpc-3 cells for overexpression of ZBTB4 and Panc-1 cells for silencing ZBTB4 expression. We used an ZBTB4 overexpression plasmid for overexpression of ZBTB4 (Fig. 7D and E) and also constructed three small interfering RNAs to silence the mRNA expression of ZBTB4 (Fig. 7F, G and H). Based on the RT-PCR results, the si-ZBTB4-1 with the highest silencing efficiency was used for the subsequent experiments and was validated by western blot The results of CCK-8 and EdU analyses showed that the proliferation ability of Bxpc-3 cells was significantly decreased after overexpression of ZBTB4 (Fig. 7I and J), and the proliferation ability of Panc-1 cells was enhanced after silencing ZBTB4 (Fig. 7K and L). Transwell analysis revealed that the overexpression of ZBTB4 significantly reduced the invasive ability of cells, and the invasive ability of cells was enhanced after silencing ZBTB4 (Fig. 7M and N).

## Discussion

Currently, tumors remain a major global public health threat, and although numerous studies have been conducted on various tumors, the mortality and reduced quality of life caused by tumors continues to cause great distress. New understanding of the immunopathogenesis of cancer resulted in the development of immunotherapy, which has achieved unprecedented success in the treatment of various cancers. Immunotherapy directed against tumors has different effects in different immune environments [26], and the cellular components and functions in the immune microenvironment influence the outcome of immunotherapy [27]; in other words, only some patients benefit from the treatment, which highlights the urgent need to identify new strategies to improve the clinical efficacy of immunotherapy.

Owing to a lack of comprehensive knowledge of ZBTB4, we used the TCGA pan-cancer database to reveal the differential expression of ZBTB4 in 26 human tumors. The results showed that ZBTB4 expression was significantly lower in many tumor tissues than in the corresponding healthy tissues. We tried to search the genomic mutation database for the cause of ZBTB4 expression downregulation, and although deep deletion of the ZBTB4 gene occurred in the genome of most tumors, the frequency of this occurrence was not significant, suggesting that genomic alteration is not one of the causes of ZBTB4 downregulation. The human protein kinase HIPK2 has a degrading effect on ZBTB4 and it can phosphorylate part of the ZBTB4 motif under normal growth conditions. Furthermore, HIPK2 increases the degradation of ZBTB4 in the state of DNA damage, which suggests that the abnormal activation of HIPK2 may be the cause of ZBTB4 downregulation in tumors [5].

We also investigated the correlation between the abnormal expression of ZBTB4 and the prognosis of cancer patients. The results of Kaplan–Meier analysis showed that ZBTB4 was associated with better overall survival and disease-specific survival, indicating that ZBTB4 is a protective factor in many cancers and has the potential to be an important cancer prognostic predictor that can influence cancer progression to some extent.

The immune microenvironment created by immune cell infiltration in tumors can directly affect the outcome of immunotherapy [28, 29], which may lead to tumor growth by blocking the onset of the immune response [30]. In this study, we found that ZBTB4 was highly correlated with immune infiltration of cancer in immune cell correlation analysis. It was significantly correlated with many immune cell infiltrations in most tumors, suggesting that ZBTB4 may influence disease progression and prognosis through the tumor immune microenvironment. In addition, we revealed a correlation between ZBTB4 and immunomodulatory factors. The results suggest that ZBTB4 may be involved in cancer progression and prognosis by interacting with the cancer microenvironment.

The total number of somatic gene coding errors, base substitutions, gene insertions or deletion errors detected per million bases is called TMB. Tissue TMB has emerged as a potential biomarker for predicting response to anti-programmed cell death 1 protein receptor (PD-1)/programmed cell death 1 protein ligand (PD-L1) therapy [31, 32]. Antibody-mediated PD-L1 blockade induces durable tumor regression and long-term disease stabilization in patients with advanced cancers, including colorectal and liver cancers [33–35].MSI also serves as a marker for the assessment of PD-1 antibody treatment efficacy and predicts prognosis in patients with selected solid tumors [36–39]. In our study, ZBTB4 was remarkably correlated with TMB and MSI in pan-cancer, and we can speculate that ZBTB4 is a powerful biomarker for predicting response to cancer immune checkpoint inhibitor blockade therapy.

In addition, we explored the molecules that ZBTB4 may affect and mapped the molecular interaction network using GeneMania. By enrichment analysis of these molecules, we discovered that ZBTB4 mainly interacts through transcription regulator activity, regulation of RNA metabolic process, negative regulation of transcription, DNA-templated, regulation of nucleobase-containing compound metabolic process and other pathways. These pathways play an essential role in tumor development [40–44]. This finding is also evidence that ZBTB4 plays an important role in tumor progression.

Even with the development of various diagnostic tools, the diagnosis of pancreatic cancer is still a serious diagnostic challenge [45, 46]. We found that the level of ZBTB4 in peripheral blood had good diagnostic performance for pancreatic cancer. Immunohistochemical staining of tissue microarrays from pancreatic cancer also clarified that there was a low expression of ZBTB4 protein in pancreatic cancer tumor tissues. This suggests that pancreatic cancer has the same deficiency of ZBTB4 expression as other cancers [7, 8, 10, 12, 13]. Further studies on cell biological functions showed that silencing of ZBTB4 significantly increased the proliferation, migration and invasion ability of pancreatic cancer cells, and overexpression of ZBTB4 in pancreatic cancer cell lines markedly inhibited the above effects. These findings suggest that ZBTB4 possesses the ability to influence pancreatic cancer progression.

In conclusion, our pan-cancer analysis of ZBTB4 indicates the presence of aberrant expression of ZBTB4 in a variety of tumors. This presence correlates with prognosis, tumor immune microenvironment and immunotherapeutic efficacy in some tumors. We also partially investigated the role of ZBTB4 in pancreatic cancer. In summary, ZBTB4 is associated with alteration of the tumor immune microenvironment, immunotherapy efficacy and tumor prognosis. It is a potential prognostic marker and a marker of tumor immunotherapy, and it may play an important role in inhibiting the progression of pancreatic cancer.

## Conclusion

In this manuscript, we show that ZBTB4 is a promising marker for cancer immunotherapy and cancer prognosis and has the potential to influence pancreatic cancer progression. The ZBTB4 belongs to the zinc finger protein family, which has a role in regulating epigenetic inheritance and is associated with cell differentiation and proliferation.

## Supplementary Information

**Additional file 1: Figure S1. **Plasmid mapping for overexpression of ZBTB4.

**Additional file 2: Figure S2. **Original image of intact gels/blots without cropping.

**Additional file 3: Table S1. **Abbreviations of cancers in the Pancancer cohort. 

**Additional file 4: Table S2. **Small interfering RNA for silencing ZBTB4.

**Additional file 5: Table S3. **Gene MANIA analysis of genes interacting with ZBTB4.

**Additional file 6: Table S4. **Gene Ontology term enrichment analysis of the ZBTB4 and its interacting genes.

**Additional file 7: Table S5. **KEGG enrichment analysis of the ZBTB4 and its interacting genes.

## Data Availability

The datasets generated or analyzed in this study are available in open access databases. In this study we used the following databases for analysis, data acquisition and visualization: TCGA ( https://portal.gdc.cancer.gov/), cBioPortal (http://www.cbioportal.org),UCSC (https://xenabrowser.net/), SangerBox 3.0 (http://vip.sangerbox.com/), TIMER ( https://cistrome.shinyapps.io/timer/), GeneMANIA (http://genemania.org/), GEO (https://www.ncbi.nlm.nih.gov/geo/). For the GEO database, we used the dataset which was coded as GSE125158 (https://www.ncbi.nlm.nih.gov/geo/query/acc.cgi?acc=GSE125158). All data are available from the corresponding author upon reasonable request.

## Abbreviations

ZBTB4: Zinc finger and BTB domain-containing protein 4
TCGA: The Cancer Genome Atlas
GEO: Gene Expression Omnibus
EMT: Epithelial-mesenchymal transition
TMB: Tumor mutational burden
MSI: Microsatellite instability
UCSC: University of California Santa Cruz
TPM: Transcripts Per Kilobase per Million mapped reads
TIMER: Tumor Immune Estimation Resource
ROC: Receiver operating characteristic
PMSF: Phenyl methyl sulfonyl fluoride
MDSC: Myeloid-derived suppressor cells
PD-1: Programmed cell death 1 protein receptor
PD-L1: Programmed cell death 1 protein ligand
CNA: Copy number alteration
Tregs: Regulatory T cells
CAF: Cancer-associated fibroblast
Eos: Eosinophil
Endo: Endothelial cell
HSC: Hematopoietic stem cell
Tfh: T cell follicular helper
γ/δT: T cell gamma delta
NKT: Nature killer T cell
AUC: Area under curve
TPR: True positive rate
FPR: False positive rate
CI: Confidence interval
