# Supplementary figures and images for: Identification of ZBTB4 as an immunological biomarker that can inhibit the proliferation and invasion of pancreatic cancer
Source: BMC Cancer. 2023 Mar 22;23:263. doi: 10.1186/s12885-023-10749-x (PMC10035130; doi:10.1186/s12885-023-10749-x)

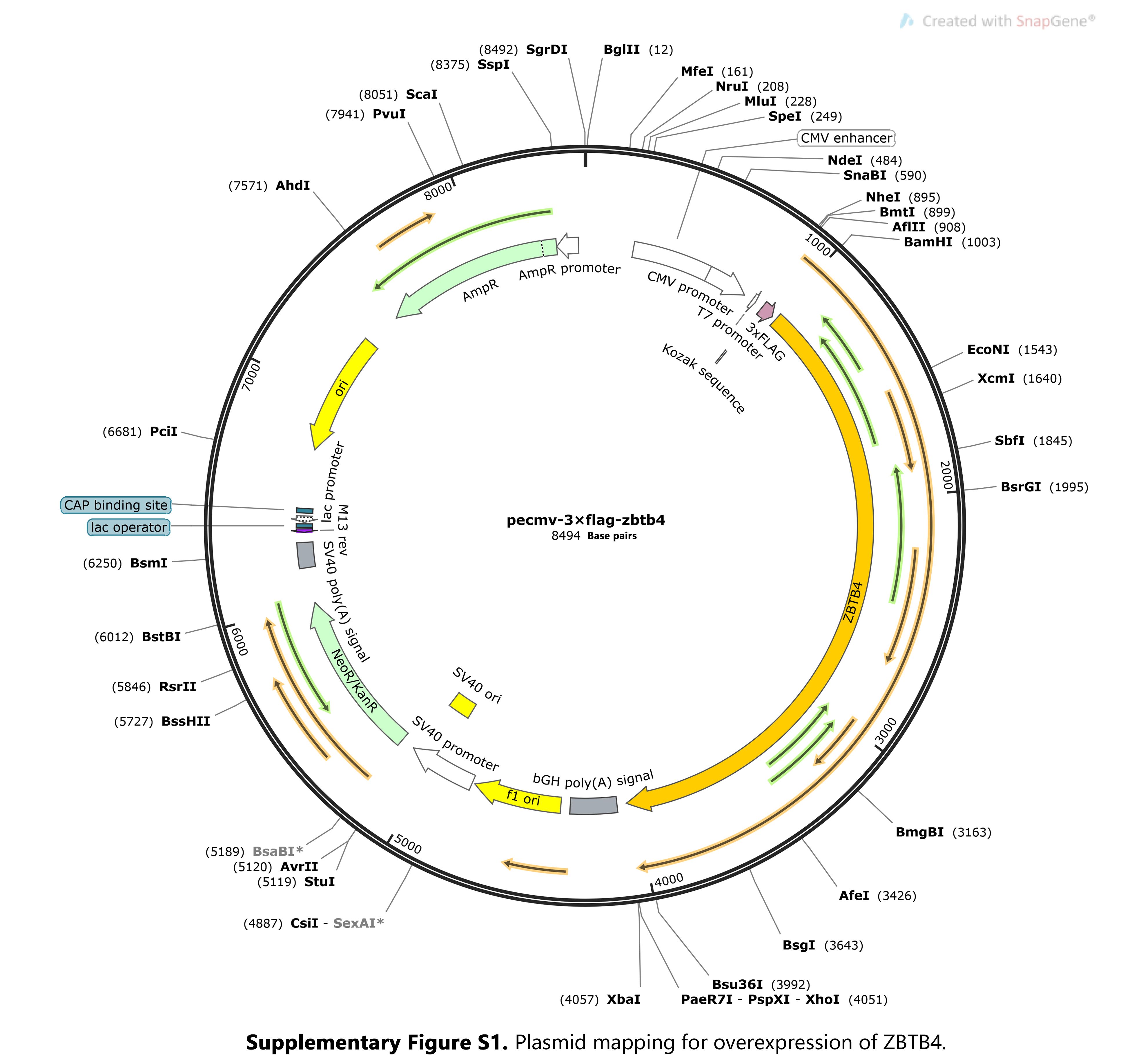

Supplement: Supplementary file 1 — Additional file 1: Figure S1. Plasmid mapping for overexpression of ZBTB4. [file 12885_2023_10749_MOESM1_ESM.jpg]
