# Supplementary material for: Identification of ZBTB4 as an immunological biomarker that can inhibit the proliferation and invasion of pancreatic cancer
Source: BMC Cancer. 2023 Mar 22;23:263. doi: 10.1186/s12885-023-10749-x (PMC10035130; doi:10.1186/s12885-023-10749-x)
Supplement: Supplementary file 2 — Additional file 2: Figure S2. Original image of intact gels/blots without cropping. [file 12885_2023_10749_MOESM2_ESM.pdf]

Figure 7A:

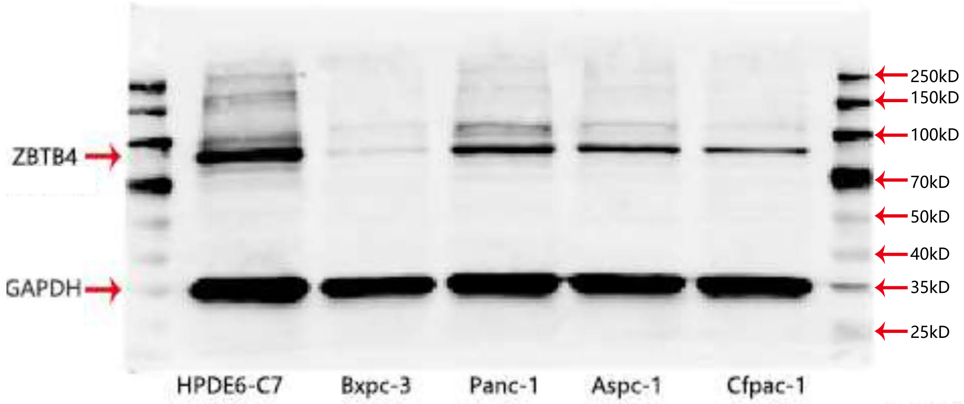

Figure 7D:

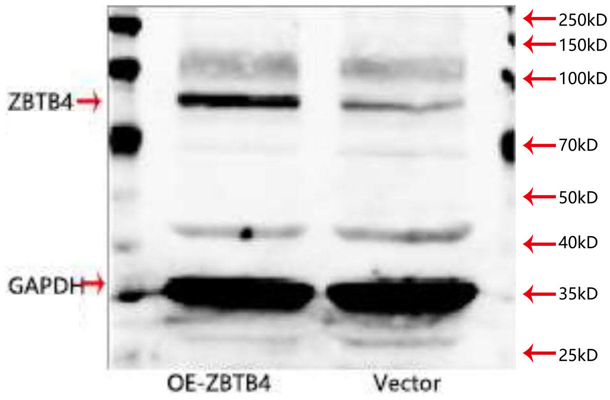

Figure 7G:

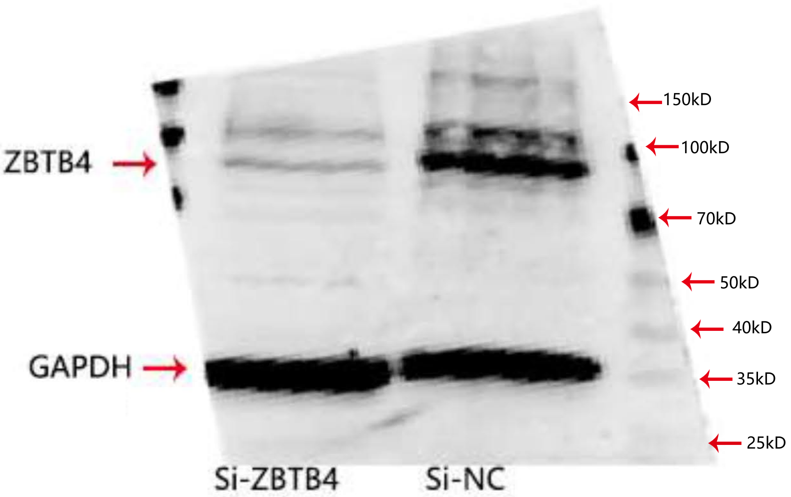

Supplementary Figure S2. Original image of intact gels/blots without cropping. The bands were visualized using the Odyssey Infrared Imaging System.
