# Supplementary material for: Identification of ZBTB4 as an immunological biomarker that can inhibit the proliferation and invasion of pancreatic cancer
Source: BMC Cancer. 2023 Mar 22;23:263. doi: 10.1186/s12885-023-10749-x (PMC10035130; doi:10.1186/s12885-023-10749-x)
Supplement: Supplementary file 3 — Additional file 3: Table S1. Abbreviations of cancers in the Pancancer cohort. [file 12885_2023_10749_MOESM3_ESM.docx]

| Supplementary Table S1. Abbreviations of cancers in the Pancancer cohort | |
| --- | --- |
| Abbreviation | Full name |
| ACC | Adrenocortical carcinoma |
| BLCA | Bladder Urothelial Carcinoma |
| BRCA | Breast invasive carcinoma |
| CESC | Cervical squamous cell carcinoma and endocervical adenocarcinoma |
| CHOL | Cholangiocarcinoma |
| COAD | Colon adenocarcinoma |
| COADREAD | Colon adenocarcinoma/Rectum adenocarcinoma Esophageal carcinoma |
| DLBC | Lymphoid Neoplasm Diffuse Large B-cell Lymphoma |
| ESCA | Esophageal carcinoma |
| FPPP | FFPE Pilot Phase II |
| GBM | Glioblastoma multiforme |
| GBMLGG | Glioma |
| HNSC | Head and Neck squamous cell carcinoma |
| KICH | Kidney Chromophobe |
| KIPAN | Pan-kidney cohort (KICH+KIRC+KIRP) |
| KIRC | Kidney renal clear cell carcinoma |
| KIRP | Kidney renal papillary cell carcinoma |
| LAML | Acute Myeloid Leukemia |
| LGG | Brain Lower Grade Glioma |
| LIHC | Liver hepatocellular carcinoma |
| LUAD | Lung adenocarcinoma |
| LUSC | Lung squamous cell carcinoma |
| MESO | Mesothelioma |
| OV | Ovarian serous cystadenocarcinoma |
| PAAD | Pancreatic adenocarcinoma |
| PCPG | Pheochromocytoma and Paraganglioma |
| PRAD | Prostate adenocarcinoma |
| READ | Rectum adenocarcinoma |
| SARC | Sarcoma |
| STAD | Stomach adenocarcinoma |
| SKCM | Skin Cutaneous Melanoma |
| STES | Stomach and Esophageal carcinoma |
| TGCT | Testicular Germ Cell Tumors |
| THCA | Thyroid carcinoma |
| THYM | Thymoma |
| UCEC | Uterine Corpus Endometrial Carcinoma |
| UCS | Uterine Carcinosarcoma |
| UVM | Uveal Melanoma |
