# Supplementary material for: Identification of ZBTB4 as an immunological biomarker that can inhibit the proliferation and invasion of pancreatic cancer
Source: BMC Cancer. 2023 Mar 22;23:263. doi: 10.1186/s12885-023-10749-x (PMC10035130; doi:10.1186/s12885-023-10749-x)
Supplement: Supplementary file 4 — Additional file 4: Table S2. Small interfering RNA for silencing ZBTB4. [file 12885_2023_10749_MOESM4_ESM.docx]

| Supplementary Table S2. Small interfering RNA for silencing ZBTB4 | | |
| --- | --- | --- |
| Serial No | Sense | Anti-Sense |
| Si-ZBTB4-1 | GCUUCAAGUCCCUUCUUCATT | UGAAGAAGGGACUUGAAGCTT |
| Si-ZBTB4-2 | GCCGCUAUUGUGAGAAAGUTT | ACUUUCUCACAAUAGCGGCTT |
| Si-ZBTB4-3 | GGGCUGCCAAAGUCACUUUTT | AAAGUGACUUUGGCAGCCCTT |
| Negative control | UUCUCCGAACGUGUCACGUTT | ACGUGACACGUUCGGAGAATT |
